# Case report: Dravet syndrome, feeding difficulties and gastrostomy

**DOI:** 10.3389/fneur.2022.993906

**Published:** 2022-09-13

**Authors:** Lisa M. Clayton, Edwina Williams, Simona Balestrini, Sanjay M. Sisodiya

**Affiliations:** ^1^Department of Clinical and Experimental Epilepsy, UCL Queen Square Institute of Neurology, London, United Kingdom; ^2^Chalfont Centre for Epilepsy, Buckinghamshire, United Kingdom; ^3^Dravet Syndrome Parent, London, United Kingdom; ^4^Department of Pediatrics, Meyer Children's University Hospital, Florence, Italy

**Keywords:** Dravet syndrome, co-morbidities, SCN1A, gastrostomy (PEG), weight loss, feeding and swallowing trouble

## Abstract

Dravet syndrome (DS) is a developmental and epileptic encephalopathy associated with variants in the voltage-gated sodium channel alpha 1 subunit (*SCN1A)* gene in around 90% of individuals. The core phenotype is well-recognized, and is characterized by seizure onset in infancy, typically with prolonged febrile seizures, followed by the emergence of multiple seizure types that are frequently drug-resistant, developmental delay, and intellectual disability. Comorbidities are common and include autism spectrum disorder, gait impairment, scoliosis, and sleep disorder. Feeding difficulties and weight loss are frequently reported by DS caregivers, and negatively impact quality of life, yet have received little attention. Here we report an adult with DS who developed reduced food and fluid intake in adolescence, resulting in weight loss and malnutrition. No underlying cause for her feeding difficulties was identified, and she subsequently required insertion of a percutaneous endoscopic gastrostomy. We review the occurrence of feeding difficulties in people with DS and discuss potential mechanisms.

## Introduction

DS is a developmental and epileptic encephalopathy. It is associated with variants in the voltage-gated sodium channel alpha 1 subunit (*SCN1A)* gene in around 90% of individuals (1), and is one of the most common monogenic epilepsies, with an incidence of 1 per 12,200 live births (2). The core phenotype of DS is well-characterized. Symptom onset is at around 6 months, typically with recurrent, prolonged, tonic clonic, or hemiclonic seizures. The initial seizures are characteristically triggered by fever (febrile seizures), and/or vaccination (1, 3). Subsequently, multiple, afebrile seizure types emerge, that are usually resistant to treatment with antiseizure medications (1, 3). Status epilepticus is common (1), and premature mortality is estimated to affect between 10 and 20% of people (4–6), most commonly due to sudden unexplained death in epilepsy (SUDEP) or status (7). Development is initially normal, but begins to slow, plateau, or regress after seizure onset, typically from 2 years of age (1, 3). Most individuals develop moderate to severe intellectual disability (1, 3, 8).

In addition to epilepsy, developmental delay, and intellectual disability, other “comorbid” conditions are common in DS, and include autism spectrum disorder (ASD) (9–12), gait impairment (9, 10, 13–16), scoliosis (12, 16), and sleep disorder (9, 16–21).

Difficulties related to feeding, and weight loss/failure to gain weight, are frequently reported in surveys of DS caregivers (9, 12, 16, 17, 22, 23). Reported feeding difficulties include appetite disturbance (9, 12, 16, 17, 22, 23), food fads and fussy/selective eating (22), prolonged meal times (22), inability to feed independently (24), and difficulty with chewing and swallowing (9, 12, 16, 22, 24). In one of the largest surveys of comorbidities in children with DS, 99% of caregivers reported at least one concern relating to feeding, making it the most frequently reported category of concern (22). Despite this, issues related to feeding and weight loss in DS have received little attention. In our single-centre experience of caring for more than 50 adults with DS, feeding difficulties and weight loss are commonly encountered, and 20% of individuals have undergone percutaneous endoscopic gastrostomy (PEG) insertion to manage these issues (Clayton, unpublished).

Here we report an adult with DS, highlighting some of the common but under-recognized issues related to feeding and PEG insertion in this population.

## Case description

Taliah was born following an uneventful pregnancy. At 2 months she had a prolonged generalized tonic clonic seizure (GTCS) 12 h after her first immunization. At 3 months, following her second immunization, she had a further prolonged GTCS. Habitual, unprovoked seizures including myoclonic jerks, unclassified unresponsive episodes (possibly absence or atypical absence seizures), and GTCS began shortly after. Seizures were drug-resistant, and she experienced several episodes of status epilepticus. Taliah's early development was normal, but after a severe and prolonged GTCS at 4 years there was developmental regression, and subsequent intellectual disability. At 10 years, Taliah was diagnosed with DS following genetic testing which identified a pathogenic stopgain variant in *SCN1A* (NM_001353948:c.G3991T:p.E1331^*^). Now in adulthood, she continues to have clusters of GTCS, occurring every 1–2 months. Taliah has ASD and thoracolumbar scoliosis, which was surgically corrected at 13 years.

**Figure 1:**
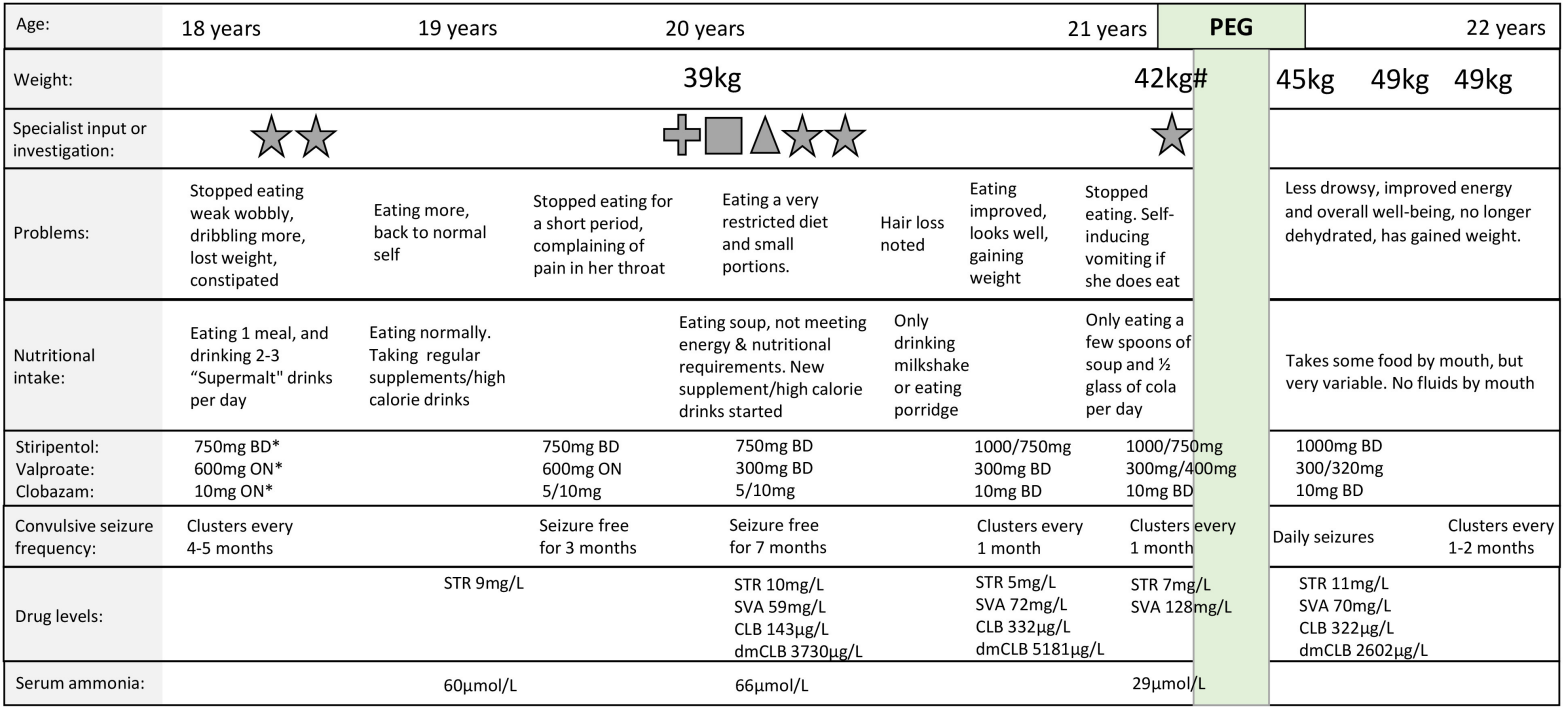
Timeline of feeding difficulties and percutaneous endoscopic gastrostomy insertion. Feeding difficulties including reduced food and fluid intake and subsequent weight loss fluctuated over 3 years prior to percutaneous endoscopic gastrostomy (PEG) insertion. # = Body mass index (BMI): 16.4 kg/m^2^; * = The combination of stiripentol (STR), valproate (SVA), and clobazam (CLB) had been prescribed for 4 years prior to symptom onset at 18 years, with no dose increases, or introduction of new medications, in the preceding 3 years; star = dietician review, cross = speech and language therapy review; square = ear nose and throat specialist review; triangle = barium swallow; Green highlight = time of PEG insertion; BD = twice daily, ON = at night; drug doses separated by a forward slash (e.g., 5/10 mg) refer to a morning and evening dose; serum therapeutic drug level ranges: stiripentol 2–22 mg/L; valproate 50–100 mg/L; clobazam 30–300 μg/L; Desmethylclobazam (dmCLB) 300–3,000 μg/L; serum ammonia normal range 11–32 μg/L. Weights prior to the age of 20 were not available.

During adolescence, Taliah's food and fluid intake began to reduce, and weight loss ensued (Figure 1). There were no clear precipitants. She had been treated with a combination of stiripentol, valproate and clobazam for 4 years prior to symptom onset, with no dose increases, or introduction of new medications, in the preceding 3 years. Assessments and investigations, including by speech and language therapy, ear nose and throat specialists, blood tests (including antiseizure drug levels and ammonia levels), and barium swallow, did not reveal a cause. Over a period of 3 years, Taliah's food intake fluctuated, with episodes lasting for weeks where she would refuse to eat or drink, and where medication compliance could be challenging. Over time, seizure-free periods which occurred between clusters of GTCS, became shorter in duration (Figure 1). The need for a PEG was discussed, but like many caregivers of children/adults with neurological disability, the decision to proceed with PEG insertion was difficult for Taliah's family (25). Overtime, weight loss and signs of malnutrition became evident, and eventually a PEG was inserted to supplement Taliah's nutrition and fluid intake, and to allow for consistent medication administration. Since undergoing the PEG insertion, Taliah's weight has increased, she has more energy and sleeps less during the day. Her overall well-being has improved, and she is more engaged, interactive, and communicative. Taliah receives all fluid, and most nutrition via the PEG, but still eats some food orally for pleasure. Mealtimes are much less stressful for her family, and medication compliance is ensured.

## Discussion

**Figure 2:**
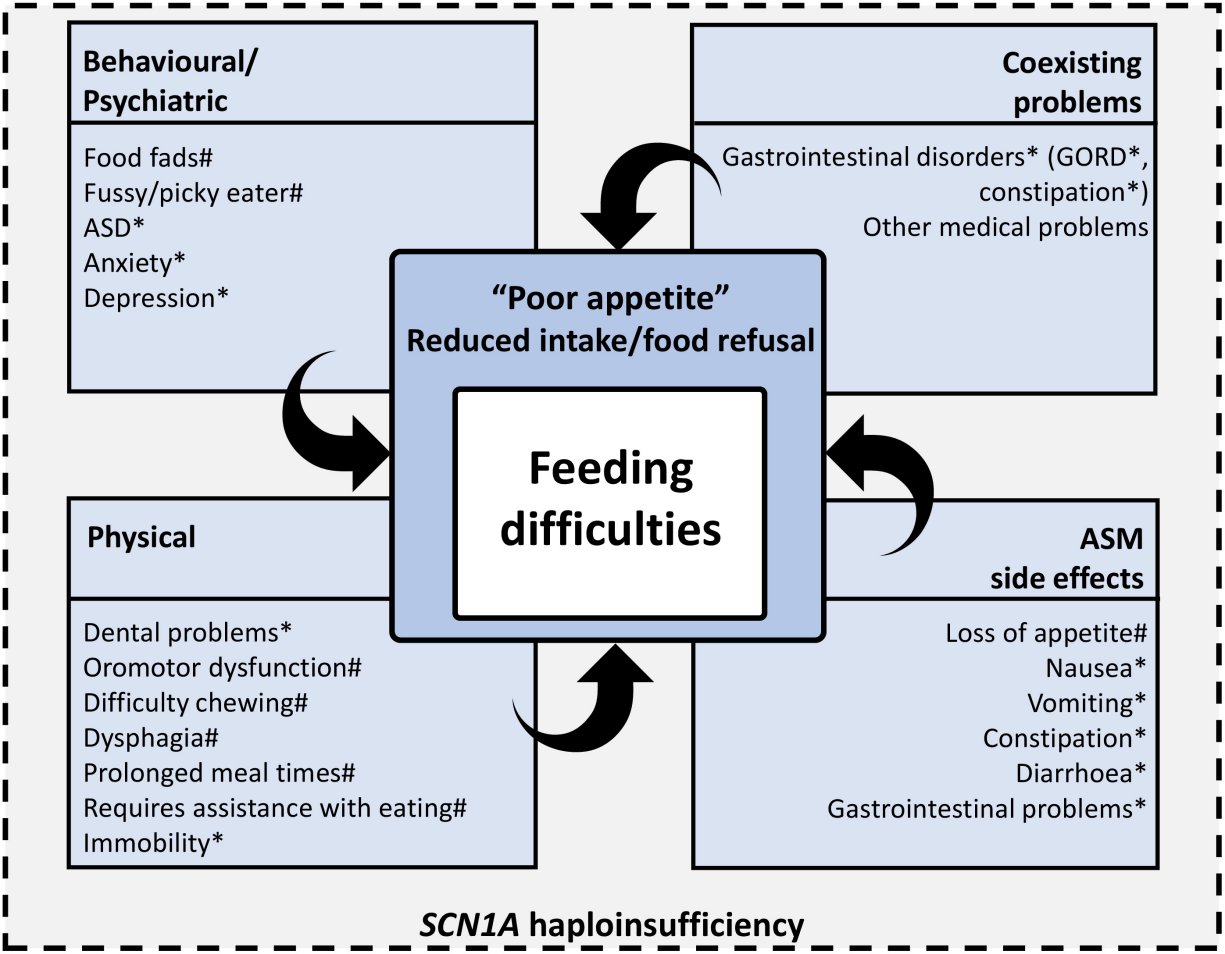
Spectrum of potential feeding difficulties in people with DS. The true spectrum of feeding difficulties in people with DS are unknown and likely reflect a combination of physical, behavioral, medication, gastrointestinal and other medical problems. These issues may directly result in feeding difficulties (i.e., dysphagia) or may lead to reduced food intake/food refusal, which is observable to caregivers and clinicians as “poor appetite.” The underlying disease biology of DS (*SCN1A* haploinsufficiency) may contribute to feeding difficulties through some of these factors. # = Feeding issue reported in caregiver surveys of people with DS, * = Problems reported in people with DS that may also contribute to feeding issues.

The true nature and spectrum of feeding difficulties in people with DS is unknown. Caregiver-reported “loss of appetite” is one of the most common feeding difficulties described in people with DS, yet the factors that lead to reduced food intake and food refusal (observed as “loss of appetite”), are likely to be complex and multifaceted. In children with neurodisability, oropharyngeal dysfunction, dental abnormalities, gastrointestinal disorders (such as gastro-esophageal reflux and constipation), pain and behavioral factors [including those related to comorbid ASD (26)], all contribute to difficulties with feeding (27–29). Many of these issues are reported in people with DS, and may contribute to reduced food intake/food refusal, and other feeding difficulties. In addition, some of the most frequently prescribed antiseizure medications in DS, including topiramate (30), stiripentol (31), cannabidiol (32), and fenfluramine (33), are commonly associated with decreased appetite, weight loss and other gastrointestinal symptoms. Feeding difficulties may also be a consequence of the underlying disease biology of DS (Figure 2).

Despite their frequency in DS, issues related to feeding have received little attention, but are important to recognize and address, as they can have a negative impact on the quality of life for both the person with DS and their caregiver (9, 17, 22). Feeding difficulties can result in reduced food intake, leading to malnutrition and its consequences, poor medication compliance, and the risk of morbidity and mortality associated with a deterioration in seizure control. In addition, oropharyngeal dysfunction can put individuals at risk of aspiration pneumonia. We would recommend that all individuals who experience feeding difficulties should be referred promptly to a dietician and speech and language therapist for formal review.

As experienced by Taliah's family, the decision to proceed with gastrostomy in children/adults with neurological disability can be difficult for caregivers (25), and it is important that adequate and appropriate information is provided to facilitate shared decision making (34). Greater awareness and understanding of feeding difficulties in DS are needed to ensure that these issues are proactively sought during clinical review, contributing factors addressed, and to facilitate earlier discussions between clinicians and caregivers around the potential need for gastrostomy. Recognition of these issues, and support from clinicians in addressing them early, should help to mitigate anxiety for caregivers around feeding, and avoid delays in intervention when necessary, minimizing the risk of malnutrition, dehydration and problems related to medication compliance.

Developmental and epileptic encephalopathies such as DS are complex conditions, often with multisystem comorbidities that extend beyond that of epilepsy. Current treatments for DS are symptomatic, predominantly aimed at controlling seizures, and do not modify underlying disease pathophysiology. Reducing seizure burden in DS has beneficial effects beyond seizure control, including improvements in cognition, language and mobility (35), but there is insufficient evidence to suggest that treatment with antiseizure medications have a direct influence on other wider disease manifestations, such as neurodevelopmental, behavioral, motor, sleep and feeding difficulties. Disease-modifying therapies, currently undergoing clinical trials, have the potential to address the full spectrum of symptoms in DS as they directly target the underlying disease pathophysiology (18). Whilst these novel therapies are undergoing development, it is important for those caring for people with DS to consider the full spectrum of comorbid conditions that occur, and ensure that all aspects of the disease are addressed where possible. Caregivers have emphasized that therapies that address the wider spectrum of comorbidities in DS (alongside seizures) will improve quality of life for people with DS and have a positive effect on family wellbeing (18).

## Parent's perspective

My daughter is now 22 years old. She has had seizures for most of her life. They started when she was 2 months old, just after an immunization. She was eventually diagnosed with epilepsy at 6 months, with the doctor at the time reassuring me that she would “grow out of it.” Throughout her early years she had so many seizures that we were practically living in the hospital. As she grew older her seizures never settled down. She has tried almost every antiseizure medication, and at one stage she was taking six different types. When she was 4 years old, she had a major seizure; she stayed in hospital for weeks, and since this episode she has never been the same again.

When Taliah was 10 years old, after having numerous tests, she was eventually diagnosed with DS. I didn't know what DS was, and I found it difficult to take everything in. There was so much information, I could not fully grasp the full extent of my daughter's condition.

Over the years she was in and out of hospital. At one point she was put into an induced coma because her seizures would not stop. That was such a difficult time as I felt useless, and I was frightened that I was going to lose my daughter. The light of my life.

Since her diagnosis, things have continued to be difficult. As Taliah grew older, I noticed that she began to lose her appetite. She has never been the best eater, and would always focus on only eating certain foods, but as she grew into a young adult, she started to refuse food completely. I tried everything to get her to eat, but she wouldn't. She complained of her throat hurting and just would not consume anything, only taking a drink now and again. She was seen by numerous specialists, and had lots of tests to find out why she did not want to eat, but all of the tests were normal, and nothing seemed to help. Then, for no clear reason, she slowly started to eat again, but only soft foods like yogurt and mashed potatoes.

By this time Taliah was an adult, and I learnt from her doctors that Taliah's eating difficulties were part of her DS. I felt relieved, but worried at the same time. I had comfort knowing that this was part of her condition, but worried, as I realized that it could get worse as she grew older. It was recommended that Taliah should have a PEG fitted, so that nutrition, fluids and medication could be given by a tube directly into her stomach. The PEG, and how it would affect Taliah, was explained to me, but at the time I was concerned about Taliah having to undergo the procedure, and because she was still eating small amounts, I did not want to put her through more distress than necessary. As a parent you always want to do the best for your child, but sometimes it is difficult to figure out what the best thing is. As time went on, Taliah was doing well, I did some research and asked questions about the PEG, and my conclusion was that she was not going to have it.

Within a year she stopped eating again, this time for 3 months. My heart sank. I was watching my daughter wither away in front of my very eyes. I didn't know what to do. Her skin changed and her hair fell out. She was starving herself again. I hated myself because I realized I had made a huge mistake. I should have gone ahead with the PEG a year ago. Now look at what I had caused. I contacted Taliah's doctors and explained what was happening, and asked if Taliah could have a PEG inserted.

The PEG procedure was promptly arranged. I was still scared about the operation, but I had to do this for Taliah, and so she had her PEG inserted. It was difficult at first as we spent almost 3 weeks in the hospital. Taliah found it difficult to accept her PEG, and she could not tolerate the required rate of feed. She had a lot of discomfort at first, and she wanted to pull it out. It was difficult to watch her being in so much pain.

Being discharged home was a relief, but also very daunting, as I now had to deal with the PEG feed on my own without the support of the nurses at the hospital. However, once we got home, I had all the equipment that I needed to use the PEG, as well as telephone numbers that I could call in case I needed support. I was so nervous at first about making mistakes, I followed all the steps and I think I did a great job!

I was supported by the PEG nurses to clean the wound and to advance and turn the PEG tube. Advancing and turning the PEG tube every week was difficult, as this was quite painful for Taliah. My first try was horrible, I was shaking, and I even cried because I hated seeing my child in pain. I didn't want to do it, but the PEG nurse reminded me how important it was to prevent the base of the tube inside the stomach from becoming stuck, which would require another operation to remove it. I had to be brave, and I did what I had to do. Overtime it got easier, and I found clever ways of turning the tube so that Taliah does not even notice what I am doing.

Having the PEG fitted for my child has been the best thing to have happened. There is no longer a worry when Taliah does not want to eat, as she has her feed every day; it has brought me peace of mind when she refuses food.

At the start of her feeds, she required a 1,000 ml bottle which took 14 h to complete, but now she only requires 500 ml which runs over 5 h. Taliah has a small rucksack which she keeps her feed and her pump in, so she can go to college and do outdoor activities easily, and feed at the same time. She is no longer dehydrated as she gets regular water flushes throughout the day. She no longer sleeps for most of the day as her body has the correct nutrients. Her hair is flowing and glossy and she is a chatter-box now!

It has been a long journey, but I can honestly say that the PEG feed has been the absolute best thing for Taliah, and a God send for our family. I would absolutely recommend anyone who needs a PEG to have one, as it really does change lives.

## Data availability statement

The raw data supporting the conclusions of this article will be made available by the authors, without undue reservation.

## Ethics statement

Ethical review and approval was not required for the study on human participants in accordance with the local legislation and institutional requirements. The patients/participants provided their written informed consent to participate in this study. Written informed consent was obtained from the individual (s) for the publication of any potentially identifiable images or data included in this article.

## Author contributions

LC, SB, and SS contributed to conception and design of the case report and discussion. LC wrote the first draft of the manuscript. EW wrote the first draft of the parent perspective. All authors contributed to manuscript revision, read, and approved the submitted version.

## Funding

The work was supported by the Epilepsy Society and Dravet Syndrome UK. The publishing fee was contributed by Stoke Therapeutics. The funder was not involved in the study design, collection, analysis, interpretation of data, the writing of this article, or the decision to submit it for publication.

## Permission statement

Taliah's family requested her name be used in this article.

## Conflict of interest

The authors declare that the research was conducted in the absence of any commercial or financial relationships that could be construed as a potential conflict of interest.

## Publisher's note

All claims expressed in this article are solely those of the authors and do not necessarily represent those of their affiliated organizations, or those of the publisher, the editors and the reviewers. Any product that may be evaluated in this article, or claim that may be made by its manufacturer, is not guaranteed or endorsed by the publisher.
